# Discovery of Small Molecules Against Foot-and-Mouth Disease Virus Replication by Targeting 2C Helicase Activity

**DOI:** 10.3390/v17060785

**Published:** 2025-05-29

**Authors:** Saisai Zhou, Suyu Mu, Shuqi Yu, Yang Tian, Sijia Lu, Zhen Li, Hao Wu, Jiaying Zhao, Huanchun Chen, Shiqi Sun, Yunfeng Song

**Affiliations:** 1 State Key Laboratory of Agricultural Microbiology, Huazhong Agricultural University, Wuhan 430070, China; zss2021302010119@webmail.hzau.edu.cn (S.Z.); yushuqi@webmail.hzau.edu.cn (S.Y.); tian_1995@webmail.hzau.edu.cn (Y.T.); lsj20010712@163.com (S.L.); swanlizen@webmail.hzau.edu.cn (Z.L.); 2023302010068@webmail.hzau.edu.cn (H.W.); z_jia_y@webmail.hzau.edu.cn (J.Z.); chenhch@mail.hzau.edu.cn (H.C.); 2 College of Veterinary Medicine, Huazhong Agricultural University, Wuhan 430070, China; 3 Lanzhou Veterinary Research Institute, Chinese Academy of Agricultural Sciences, Lanzhou 730046, China; mutou6633937@126.com (S.M.); sunshiqi@caas.cn (S.S.)

**Keywords:** FMDV, 2C protein, helicase, antiviral inhibitor

## Abstract

Background: The 2C protein of foot-and-mouth disease virus (FMDV), a member of helicase superfamily 3 (SF3), drives viral genome replication and serves as a critical target for antiviral drug development. Methods: A fluorescence resonance energy transfer (FRET)-based high-throughput screening (HTS) platform was developed to identify 2C helicase inhibitors. Primary screening evaluated 4424 compounds for helicase inhibition. Molecular docking analyzed inhibitor interactions with the N207 residue within the catalytic core and helicase inhibition assays classified the inhibitor type (mixed, competitive, noncompetitive). Differential scanning fluorimetry (nanoDSF) quantified 2C thermal destabilization. Antiviral activity was assessed via indirect immunofluorescence, RT-qPCR, and plaque reduction assays. Results: Six compounds inhibited 2C helicase activity at >620 μM. Molecular docking revealed hydrogen bonding, hydrophobic interactions, and π-cation stabilization at the catalytic core. 2-MPO and MPPI were classified as mixed-type inhibitors, 5-TzS^−^ and 2-PyOH as competitive, and DCMQ/Spiro-BD-CHD-dione as noncompetitive. NanoDSF showed a Δ*T_m_* ≥ 1.5 °C (2.5 mM compounds), with reduced destabilization in N207A mutants. Antiviral assays identified 2-MPO and MPPI as optimal inhibitors. MPPI achieved effective FMDV suppression at 160 μM, exhibiting two orders of magnitude higher potency than 2-MPO (400 μM). Conclusions: The established FRET-based HTS platform targeting 2C helicase facilitates anti-FMDV lead discovery, while 2C inhibitors may serve as an effective therapeutic strategy against other picornaviruses.

## 1. Introduction

Foot-and-mouth disease (FMD) is a highly contagious viral infection causing severe economic losses in cloven-hoofed livestock. The causative agent, FMDV, belongs to the genus *Aphthovirus* within the family *Picornaviridae* [1,2,3]. FMDV comprises seven antigenically distinct serotypes (A, O, C, Asia 1, SAT 1, SAT 2, and SAT 3), complicating vaccine development. The FMDV open reading frame (ORF) encodes a polyprotein precursor. Proteolytic cleavage generates four structural proteins (VP1–VP4) and ten nonstructural proteins (NSPs) including L^pro^, 2A, 2B, 2C, 3A, 3B_1–3_, 3C^pro^, and 3D^pol^. Transient cleavage intermediates derived from this process regulate viral replication and host immune evasion [3].

The 2C protein is a phylogenetically conserved nonstructural protein across *Picornaviridae* [4]. SF3 members are viral hexameric helicases, the with AAA+ ATPase catalytic domains critical for nucleotide hydrolysis [5,6]. The picornaviral 2C protein exhibits multifunctional activities including RNA-binding, ATPase, ribonuclease, helicase, and RNA chaperone activities. Its ATPase domain, constituted by Walker A/B motifs, Motif C, and an arginine finger (Arg-finger), forms the structural core essential for nucleotide hydrolysis [5,7]. While most picornaviral 2C proteins lack canonical helicase activity, unconventional RNase IV-like activity has been identified in hepatitis A virus (HAV) 2C [8]. Enterovirus 71 (EV71) 2C displays ATP-independent helicase and RNA chaperone activities, facilitating RNA structural remodeling during replication [9]. Similar ATP-independent helicase activity was confirmed in coxsackievirus B3/5 (CVB3/5) homologs [10]. FMDV 2C demonstrates intrinsic helicase activity in vitro, with biochemical assays validating its ribonuclease specificity and RNA chaperone function [11].

The 2C protein is critical for viral replication and pathogenesis in FMDV, with conserved structural features driving novel strategies [7]. Structural studies have revealed a conserved C-terminal α-helix near the ATP-binding cleft of adjacent 2C monomers. This helix occupies a hydrophobic pocket, enabling intermolecular interactions essential for ATPase activation and viral replication [7,12]. C-terminal analyses identified a zinc finger-like motif adjacent to a pocket-binding loop (PBL), which mediates enteroviral replication compartment assembly via lipid droplet interactions [13]. Picornaviral 2C homologs regulate both early (viral entry/uncoating) and late (virion assembly) replication stages through ATPase activity [5,6,7,14]. Recent crystallographic breakthroughs elucidated N-terminal truncated 2C structures (lacking NTD) from multiple *Picornaviridae* members, including FMDV, poliovirus (PV), HAV, CVB3, EV71, and encephalomyocarditis virus (EMCV), highlighting conserved tertiary architecture across genera [6,7,8,12,15,16]. The FMDV 2C protein plays multifaceted roles in viral replication and host immune evasion, highlighting its critical function in FMDV pathogenesis. The FMDV 2C helicase functions as a core component in FMDV replication through oligomerization, ATPase activity, modulation of the host cellular environment, and synergistic interactions with other proteins [7,14,17]. Drug design targeting 2C PBL-peptide not only effectively suppresses viral activity, but also underscores its evolutionary conservation in viruses, thereby establishing a theoretical framework for developing broad-spectrum therapeutics against picornaviruses [7]. Small-molecule inhibitors targeting 2C offer therapeutic potential by disrupting viral replication machinery. While picornaviral 2C inhibitors exist, none specifically target FMDV. This study established a helicase activity-based HTS platform to identify FMDV-specific 2C inhibitors, aiming to block viral proliferation and provide a chemical framework for developing broad-spectrum antivirals with improved specificity.

## 2. Materials and Methods

### 2.1. Cells and Viruses

BHK-21 cells (Baby Hamster Kidney fibroblasts, Pricella Biotechnology, Wuhan, China) were cultured in Dulbecco’s modified Eagle medium (DMEM; servicebio, Wuhan, China) supplemented with 10% fetal bovine serum (FBS), 100 U/mL penicillin, and 100 μg/mL streptomycin (all from servicebio, China) under standard incubation conditions (37 °C, 5% CO_2_, humidity). BHK-21 were preserved in our laboratory. The FMDV serotype O strain O/China was archived at the World Organization for Animal Health (WOAH)-designated National Foot-and-Mouth Disease Reference Laboratory (Lanzhou, China). Viral propagation was performed in BHK-21 cell monolayers, and viral titers were quantified by the 50% tissue culture infectious dose (TCID_50_) endpoint-dilution assay following established virological protocols.

### 2.2. Establishment of Screening Methods for Inhibitors of 2C Protein Helicase Activity

A FRET-based screening strategy was developed to identify 2C helicase inhibitors. Complementary HEX- and BHQ-1-labeled DNA strands were synthesized (Sangon Biotech, Shanghai, China), along with an unlabeled competitor strand (5′–TGGTGCTCGAACAGTGACTAGC–3′) to prevent non-specific binding. HEX-labeled DNA (5′-HEX-GCTAGTCACTGTTCGAGCACCA-3′) and BHQ-1-labeled DNA (5′-BHQ1-TAGATAGCCATAGATAGCATTGGTGCTCGAACAGTGACTAGC-3′) were synthesized as complementary strands. HEX/BHQ-1-labeled dsDNA (1:1.2 molar ratio) was annealed by heating to 95 °C for 3 min, followed by gradual cooling to 25 °C. Annealing efficiency was determined by fluorescence quantification (excitation/emission: 530/580 nm). Reactions containing 20 µM 2C, 0.2 μM dsDNA substrate, and 50 mM HEPES (pH 7.5) were incubated at 37 °C in 384-well plates. Key parameters were optimized including ATP (0–0.8 mM), Mg^2+^ (0–25 mM), 2C helicase (0–20 μM), dsDNA molar ratio (1:1–1:5), and additional divalent cations (Cu^2+^, Ca^2+^, Mn^2+^, Zn^2+^, Mg^2+^; 25 mM each). Fluorescence intensity (excitation/emission: 530/580 nm) was recorded at 1-min intervals for 90 min using a VICTOR Nivo plate reader (PerkinElmer) in time-resolved mode.

### 2.3. HTS Assay of 2C Helicase Activity

Screening was performed in 384-well black plates (Greiner). A custom library of 4424 commercial compounds (Specs, Zoetermeer, The Netherlands) was stored at −80 °C until use. The helicase assay buffer (filter-sterilized) comprised 50 mM HEPES (pH 7.5), 50 mM NaCl, 20 mM MgCl_2_, 1 mM DTT, 100 µM ATP, and 20 U RNase inhibitor (Thermo Fisher Scientific, Waltham, MA, USA). Purified 2C helicase (20 µM) and compounds (5 mM) were transferred into plates and incubated at 37 °C for 30 min. Baseline fluorescence (*F*_0_; excitation/emission: 530/580 nm) was recorded using a VICTOR Nivo plate reader (PerkinElmer, Waltham, MA, USA) in time-resolved fluorescence mode. The substrate (0.2 µM) was added and mixed by gentle agitation, followed by incubation at 37 °C for 1.5 h. Percentage inhibition was calculated using the formula: [1 − (*F_t_* − *F*_0_)_sample_/*(F_t_* − *F*_0_)_control_ ] × 100, where *F*_0_ and *F_t_* represent the baseline and terminal fluorescence, respectively.

### 2.4. Enzyme Inhibition Kinetic Assay

The 2C helicase activity assay employed dsDNA concentrations ranging from 80 to 320 nM, with inhibitors (0–5 mM) adjusted proportionally to the dsDNA levels and the fluorescence-based inhibition rates were calculated. Kinetic parameters (*V*_max_ and *K_m_*) were determined by fitting the data to the Michaelis–Menten equation using nonlinear regression analysis. Lineweaver–Burk plots (1/*V*_max_ vs. 1/[*S*]) were analyzed to classify the inhibition type, with triplicate measurements ensuring reproducibility.

### 2.5. Gel-Based Helicase Inhibitor Assays

The helicase activity assay was conducted by designing HEX-labeled and BHQ-1-quenched oligonucleotide single strands, following the published protocol. These strands were annealed to form the dsRNA substrate, as previously described [9,11], and analyzed using the Typhoon 5 system. HEX-labeled RNA (5′-HEX-GCUAGUCACUGUUCGAGCACCA-3′) and complementary RNA (5′-UAGAUAGCCAUAGAUAGCAUUGGUGCUCGAACAGUGACUAGC-3′) were synthesized by Sangon Biotech (China). HEX-labeled RNA and complementary RNA were annealed at a 1:1.2 molar ratio (heated to 95 °C for 3 min, then cooled to 25 °C) to generate 5′-overhang dsRNA. The reaction buffer contained 50 mM HEPES (pH 7.5), 50 mM NaCl, 20 mM MgCl_2_, 100 µM ATP, 1 mM DTT, and 20 U RNase inhibitor. Purified 2C helicase (20 µM) was pre-incubated with inhibitors (0–5 mM) in PCR tubes at 37 °C for 30 min. The dsRNA (0.2 µM) was added, and reactions (10 µL total volume) were mixed by gentle vortexing and incubated at 37 °C for 60 min. Reactions were terminated with 1 µL stop buffer (1.2 mg/mL proteinase K, 1% SDS), then mixed with 1.1 µL RNA loading buffer, and analyzed by 15% native PAGE at 100 V for 90 min in Tris-borate-EDTA buffer. Gels were imaged using a Typhoon 5 system (GE Healthcare, Chicago, IL, USA).

### 2.6. NanoDSF Analysis of FMDV 2C Protein–Ligand Interactions

Protein thermal stability (melting temperature, *T_m_*) was determined by nanoDSF, which monitors intrinsic fluorescence changes during thermal denaturation [18]. Purified 2C protein (2 mg/mL in 50 mM HEPES pH 7.5, 50 mM NaCl) was analyzed with a Prometheus NT.48 instrument (NanoTemper, Munich, Germany). Protein-compound mixtures at final concentrations of 0.25 mM and 2.5 mM were incubated for 30 min at 25 °C before loading into standard capillaries. Intrinsic fluorescence (330–350 nm emission; 280 nm excitation) was monitored during thermal ramping from 15 to 95 °C at a rate of 1.5 °C/min. *T_m_* values were determined from the first derivative maxima of fluorescence (*dF*/*dT*) using PR.ThermControl software (v6.0, NanoTemper). Ligand-induced thermal shift (Δ*T_m_*) was calculated as |*T_m_*^compound^ − *T_m_*^apo^|, where *T_m_*^apo^ represents the melting temperature of the ligand-free protein.

### 2.7. Cytotoxicity Assay

BHK-21 cells were seeded in 96-well black microplates (1 × 10^4^ cells/well) in DMEM supplemented with 10% FBS and cultured until reaching 80% confluency (12 h). Compounds (0–500 μM) were added to triplicate wells and incubated for 48 h. Cell viability was assessed using the CellTiter-Glo^®^ 2.0 assay (Promega) according to the manufacturer’s protocol. According to the manufacturer’s instructions, 100 μL of reconstituted reagent (1:1 *v*/*v*) was added to each well after a 30 min equilibration period at 25 °C. After 10 min of shaking incubation, luminescence (RLU) was measured using an EnVision^®^ reader. CC_50_ was calculated via nonlinear regression.

### 2.8. Viral Inhibition Assay

FMDV inhibitory activity was evaluated via plaque reduction, indirect immunofluorescence (IFA), and qRT-PCR. Cells seeded in 24-well plates reached 80% confluency before infection (MOI = 0.1, 1.5 h). After washing the cells with PBS, serially diluted compounds in virus maintenance medium were added. After 36 h incubation, supernatants were processed for the plaque assay, while cells were either fixed with methanol for IFA or lysed with TRIzol^®^ reagent for viral RNA quantification by qRT-PCR with virus-specific primers.

### 2.9. Indirect Immunofluorescence Assay

BHK-21 cells infected with FMDV were fixed with pre-chilled methanol for 10 min at −20 °C. After three PBS washes (5 min each), cells were permeabilized with 0.5% Triton X-100 for 30 min and then blocked with 2.5% bovine serum albumin (BSA) in PBS for 30 min at room temperature (RT). Following additional PBS washes, cells were incubated with rabbit anti-FMDV polyclonal antibody (1:500 dilution in PBS) for 1 h at RT. Unbound antibodies were removed by triple PBS washing (5 min each), followed by incubation with Alexa Fluor 488-conjugated Goat Anti-Rabbit IgG (1:500 dilution) for 30 min at RT. After three PBS washes, the nuclei were counterstained with 4′,6-diamidino-2-phenylindole DAPI (1 μg/mL) for 5 min at RT. Finally, fluorescence images were acquired using an EVOS FL Auto Imaging System (Thermo Fisher Scientific) after three final PBS washes.

### 2.10. Plaque Assay

Supernatants harvested from FMDV-infected cell cultures were subjected to 10-fold serial dilutions (10^−1^–10^−6^) in DMEM maintenance medium [7]. Diluted viral suspensions were added to confluent BHK-21 monolayers in 24-well plates (triplicate wells per dilution) and incubated at 37 °C under 5% CO_2_. After a 1.5 h adsorption at 37 °C, cells were washed three times with PBS and overlaid with DMEM containing 3% carboxymethylcellulose (servicebio, China), 1% penicillin-streptomycin, and 1% FBS. After 48 h of incubation, cells were fixed with 10% neutral-buffered formalin for 60 min at RT, washed three times with PBS, and stained with 0.5% crystal violet in methanol/acetone (1:3 *v*/*v*) for plaque visualization. Plaque counts were manually enumerated under a stereomicroscope.

### 2.11. Real-Time qPCR

The total RNA was isolated from cultured cells using AIDzol Total RNA Extraction Reagent (Aidlab Biotechnologies Co., Ltd., Beijing, China) according to the manufacturer’s protocol. FMDV RNA quantification was performed by RT-qPCR. The qPCR amplification was conducted with the HiScript II One Step RT-PCR Kit (Vazyme Biotech, Nanjing, China). Virus-specific primers for FMDV were designed as follows: Upstream primer: 5′–ACTGGGTTTTACAAACCTGTGA–3′; Downstream primer: 5′–GCGAGTCCTGCCACGGA–3′; Probe: 5′–6–FAM–TCCTTTGCACGCCGTGGGAC–BHQ-1–3′.

### 2.12. Statistical Analysis

All experiments included biological and technical triplicates. Data represent the mean of three biological replicates. Statistical significance (*p* < 0.05) was determined by one-way ANOVA using Tukey’s HSD test.

## 3. Results

### 3.1. Optimization of 2C Helicase Enzymatic Activity

**Figure 1:**
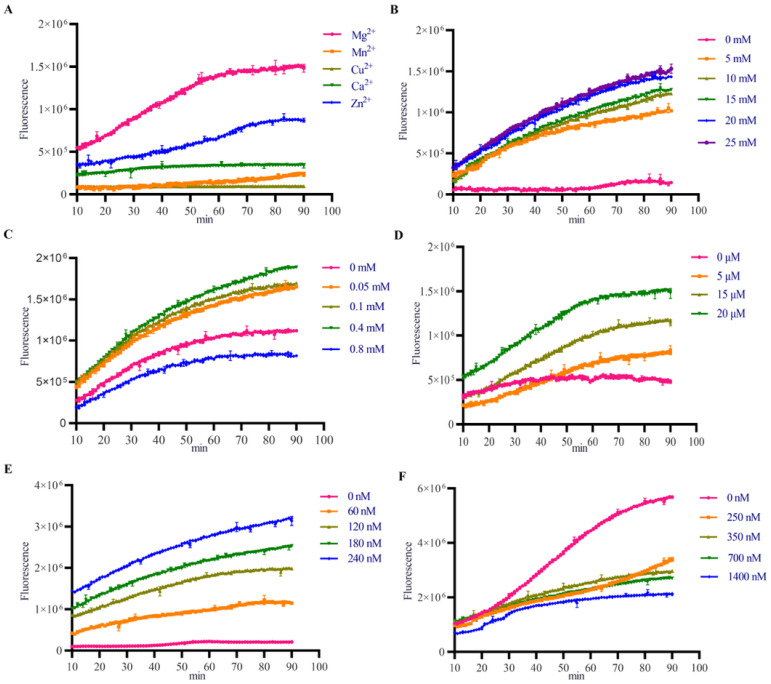
Effect of different conditions on 2C helicase activity. (**A**) Effect of divalent metal ions on 2C helicase activity. (**B**) Effect of Mg^2+^ concentration on 2C helicase activity. (**C**) ATP concentration-dependent helicase activity of 2C protein. (**D**) Protein concentration effect on 2C helicase activity. (**E**) Substrate (dsDNA) concentration-dependent helicase activity. (**F**) Effect of competitor DNA strand concentration on 2C helicase activity.

This study utilized a FRET-based assay to quantify the dsDNA unwinding activity of FMDV 2C helicase in vitro. The results showed that no helicase activity was detected in buffers containing Mn^2+^, Ca^2+^, and Cu^2+^, while Mg^2+^ uniquely enabled helicase function (Figure 1A). Helicase activity was detected at 5 mM Mg^2+^ and increased progressively in a Mg^2+^-dependent manner up to 25 mM (Figure 1B). The dsDNA unwinding occurred under both ATP-supplemented (0–0.8 mM) conditions and ATP-free conditions. However, enzymatic efficiency was significantly reduced at 0.8 mM ATP compared with ATP-free controls (Figure 1C). Complete dsDNA unwinding was achieved at 20 μM 2C helicase, with activity linearly correlated with protein concentration (Figure 1D). The fluorescence intensity increased proportionally with rising substrate concentrations, and at 120 nM, the substrate was fully unwound after 90 min of reaction, beyond which no further time-dependent enhancement occurred (Figure 1E). Helicase efficiency inversely correlated with competing strand DNA (0–1400 nM), peaking in its absence (Figure 1F). These results establish Mg^2+^ (5–20 mM) as essential for FMDV 2C helicase activity, while ATP and competing strands are nonessential for dsDNA unwinding.

### 3.2. HTS for 2C Helicase Inhibitors

**Figure 2:**
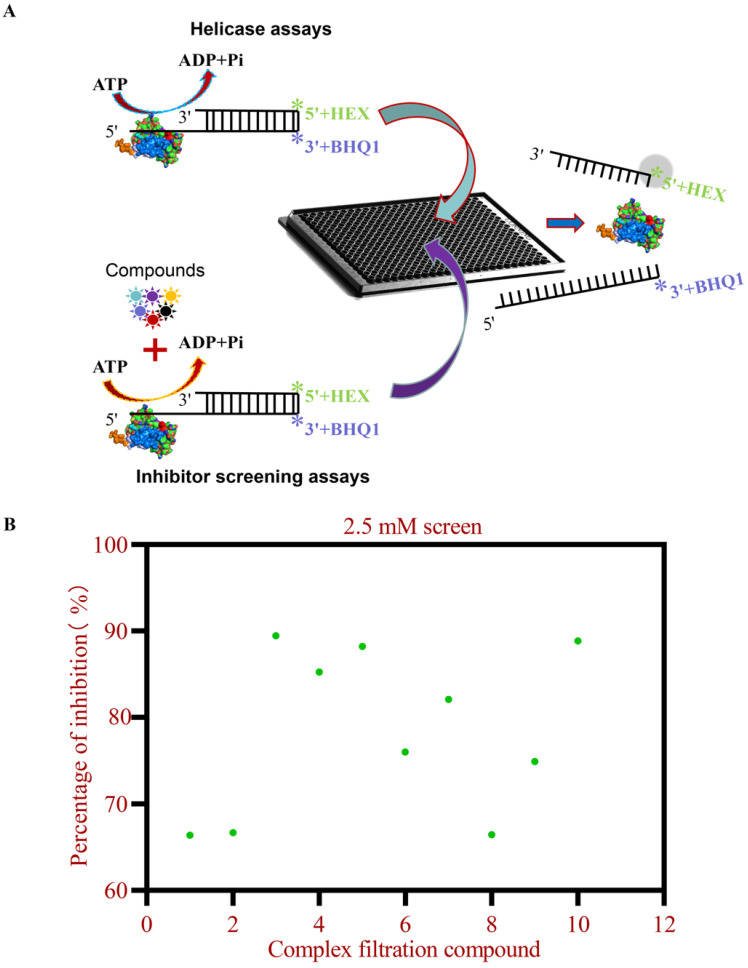
HTS for inhibitors of FMDV 2C helicase. (**A**) Schematic representation of a customized compound library screened for 2C inhibitors using a FRET-based helicase assay in a 384-well plate format. Green asterisks (*) represent the HEX fluorophore; purple asterisks (*) denote the BHQ1 quencher. (**B**) Compounds with high inhibition from the initial screening results were re-screened utilizing 2.5 mM (inhibition rate > 60%).

It has been reported that HTS methods have been extensively applied in screening for inhibitors across various antiviral drug discovery programs [18,19,20]. We developed the first in vitro FRET-based assay to characterize FMDV 2C helicase activity (Figure 2A). This assay relies on fluorescence quenching: HEX-labeled ssDNA shows suppressed emission when bound to BHQ-1-conjugated complementary strands. helicase-mediated unwinding restores fluorescence proportionally. Primary screening of 4424 compounds (5 mM) in 384-well plates identified 190 candidates showing >95% helicase inhibition compared with DMSO-treated controls. Secondary screening (2.5 mM) prioritized 10 molecules (>60% inhibition rate) based on potency, structural novelty, and pharmacophore complementarity (Figure 2B). Six lead compounds demonstrating superior inhibitory profiles were subsequently advanced for structure-activity relationship analysis and antiviral investigation (Table 1).

### 3.3. Analysis of 2C Protein-Ligand Interactions Based on NanoDSF

**Figure 3:**
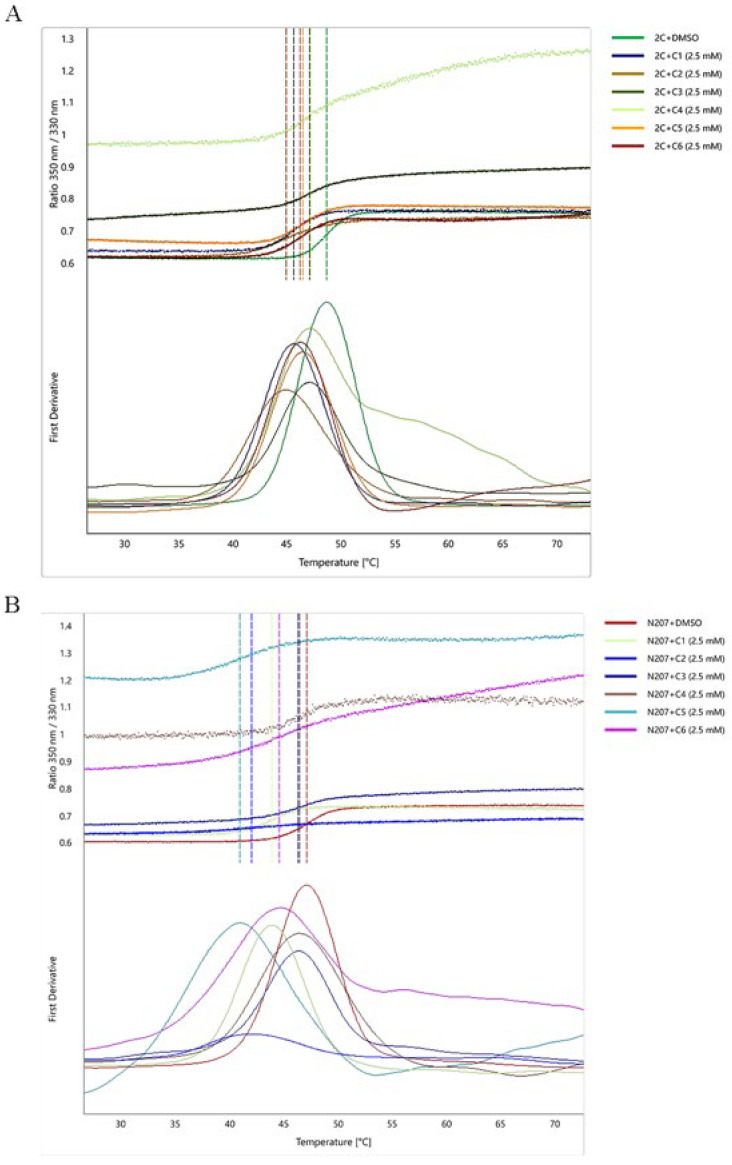
Analysis of *T_m_* value changes in the interaction of small molecule ligands with 2C and mutant N207A. (**A**,**B**) The upper half is the 350 nm/330 nm ratio after the endogenous fluorescence change during thermal stabilization. The lower half is the first order derivative corresponding to the upper half, and the temperature corresponding to the vertical line at the inflection point is the *T_m_* value. (**A**) Thermal stability profiles of 2C protein interacting with six small molecule compounds (2.5 mM). (**B**) Thermal stability profile of mutant protein N207A interacting with six small molecule compounds (2.5 mM).

NanoDSF, with its minimal sample requirements and high-throughput capability, is widely applied in ligand-binding characterization and biotherapeutic formulation screening [21,22,23]. To validate small-molecule inhibitor binding to the 2C helicase, we purified the 2C protein and incubated it with inhibitors at varying concentrations. Incubation of 2C proteins with compounds at 2.5 mM induced measurable *T_m_* shifts (Δ*T_m_* ≥ 1.5 °C) (Figure 3A). In addition, molecular docking analysis revealed that all six small-molecule compounds interact with the critical residue N207 residue within the 2C helicase structure (Figure S1). Therefore, we purified the 2C mutant protein N207A and incubated it with 2.5 mM of the compound and found that the *T_m_* value was significantly lower compared with the unmutated 2C protein (Figure 3B). Incubation of 2C proteins with compounds at 2.5 mM induced measurable *T_m_* shifts (Δ*T_m_* ≥ 1.5 °C), while lower concentrations (0.25 mM) paradoxically increased *T_m_* values versus higher doses (Figure S2). All compounds decreased *T_m_* values, contrasting with the ligand-induced stabilization typically observed in protein-ligand interactions (Figure 3). Site-directed mutagenesis at N207 significantly attenuated compound binding (Δ*T_m_*^mutant^ < Δ*T_m_*^wild−type^), confirming that this residue serves dual functions as both a helicase catalytic center and ligand-binding domain. These findings suggest that small molecules destabilize the 2C helicase, potentially through structural perturbations involving residue N207.

### 3.4. Enzyme Inhibition Kinetics Profiling

**Figure 4:**
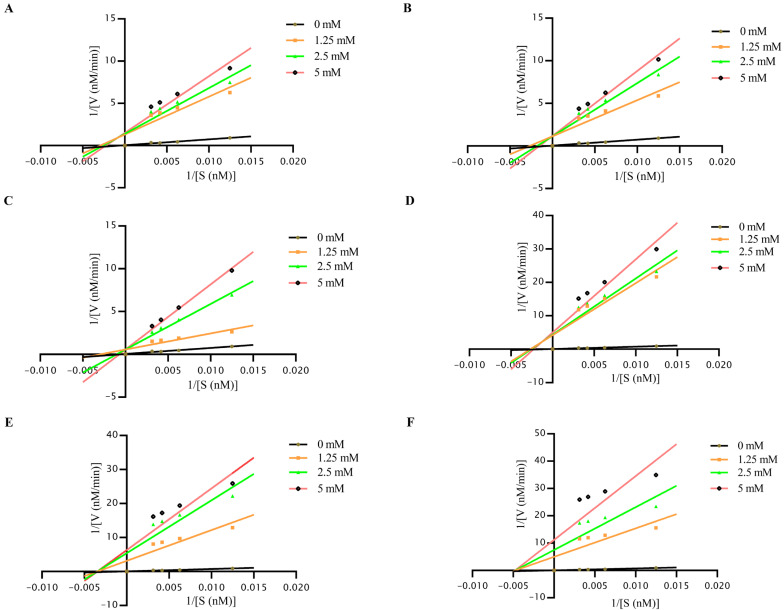
Lineweaver-Burk plot analysis of inhibition of 2C helicase activity. (**A**) The linearity between 1/V and 1/S for 2-MPO small molecule at 0, 1.25, 2.5 and 5 mM concentrations, respectively. (**B**) The linearity between 1/V and 1/S for 5-TzS^−^ small molecule at 0, 1.25, 2.5 and 5 mM concentrations, respectively. (**C**) The linearity between 1/V and 1/S for 2-PyOH small molecule at 0, 1.25, 2.5 and 5 mM concentrations, respectively. (**D**) The linearity between 1/V and 1/S for MPPI small molecule at 0, 1.25, 2.5 and 5 mM concentrations, respectively. (**E**) The linearity between 1/V and 1/S for DCMQ small molecule at 0, 1.25, 2.5 and 5 mM concentrations, respectively. (**F**) The linearity between 1/V and 1/S for Spiro-BD-CHD-dione small molecule at 0, 1.25, 2.5 and 5 mM concentrations, respectively.

To determine the inhibitor types of six small-molecule compounds, we systematically varied substrate (80–320 μM) and inhibitor (0–5 mM) concentrations, analyzing Michaelis-Menten kinetics and Lineweaver-Burk double reciprocal plots using a multimode plate reader. 2-MPO and MPPI showed substrate-dependent velocity attenuation with increased *K_m_*, their Lineweaver-Burk plots converging in the second quadrant (Figure 4A,D), aligning with mixed inhibition via dual binding. 5-TzS^−^ and 2-PyOH maintained near-constant V_max_ but elevated *K_m_*, showing parallel y-axis intercepts (Figure 4B,C), indicative of competitive inhibition. Conversely, DCMQ and Spiro-BD-CHD-dione exhibited dose-dependent *V*_max_ reduction without *K_m_* changes, with x-axis convergence (Figure 4E,F), demonstrating noncompetitive inhibition.

### 3.5. Determination of Half Cytotoxic Concentration (CC_50_) of Small Molecule Compounds

**Figure 5:**
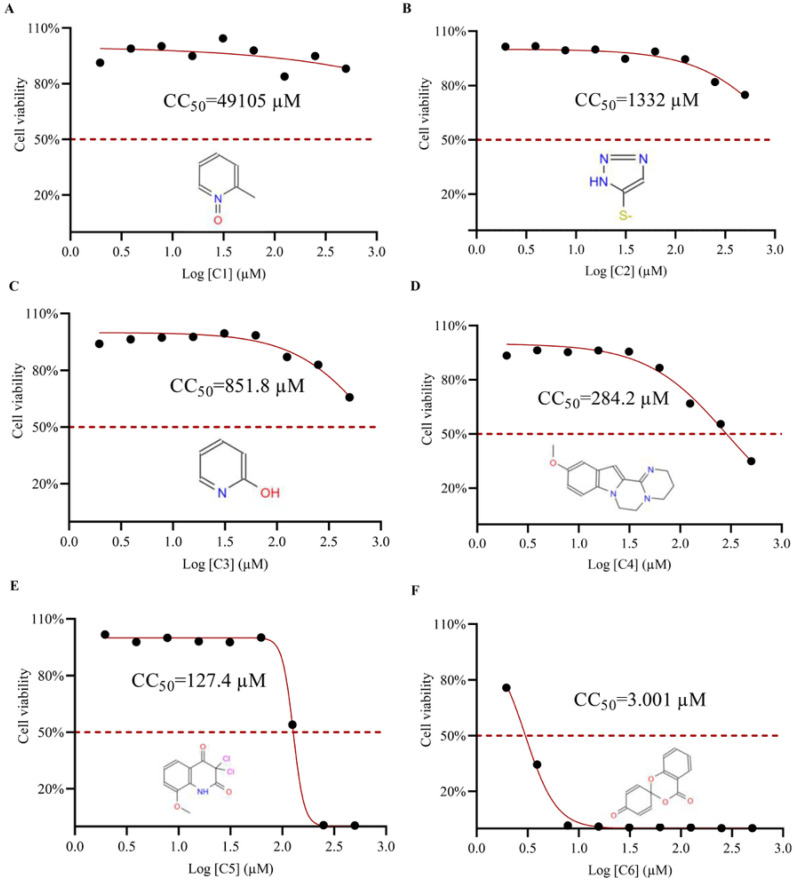
Analysis of small molecule compounds on BHK-21 cytotoxicity. (**A**) The toxicity analysis of BHK-21 by 2-MPO. (**B**) The toxicity analysis of BHK-21 by 5-TzS^−^. (**C**) The toxicity analysis of BHK-21 by 2-PyOH. (**D**) The toxicity analysis of BHK-21 by MPPI. (**E**) The toxicity analysis of BHK-21 by DCMQ. (**F**) The toxicity analysis of BHK-21 by Spiro-BD-CHD-dione. The horizontal coordinate corresponding to the intersection of the red dashed line with the solid line represents the logarithmic value of the corresponding inhibitor concentration at 50% cell activity. The portion under the red dashed line shows the 2D structure of the small molecule compound.

In pharmacological safety assessment, CC_50_ is a critical parameter defining the therapeutic index and safe dosage ranges [18,24]. To evaluate BHK-21 cell toxicity induced by 2C helicase inhibitors, we employed the CellTiter-Glo Assay kit to quantify intracellular ATP levels, followed by nonlinear regression analysis. Dose-response curves spanning nine concentrations (1.95–500 µM) demonstrated low cytotoxicity for 2-MPO, 5-TzS^−^ and 2-PyOH, with cell viability exceeding 50% at the highest tested concentration (500 µM). 2-MPO exhibited exceptional biocompatibility in BHK-21 cells (CC_50_ > 10 mM) (Figure 5A). In contrast, spiro-BD-CHD-dione showed marked cytotoxicity (CC_50_ = 3.001 μM), representing the most toxic compound (Figure 5F). Following antiviral safety thresholds (low-risk: CC_50_ > 100 μM; high-risk: CC_50_ < 20 μM), spiro-BD-CHD-dione exceeded toxicity limits and was excluded, while C1-C5 met low-toxicity criteria for preclinical development.

### 3.6. Antiviral Effect in Cells

**Figure 6:**
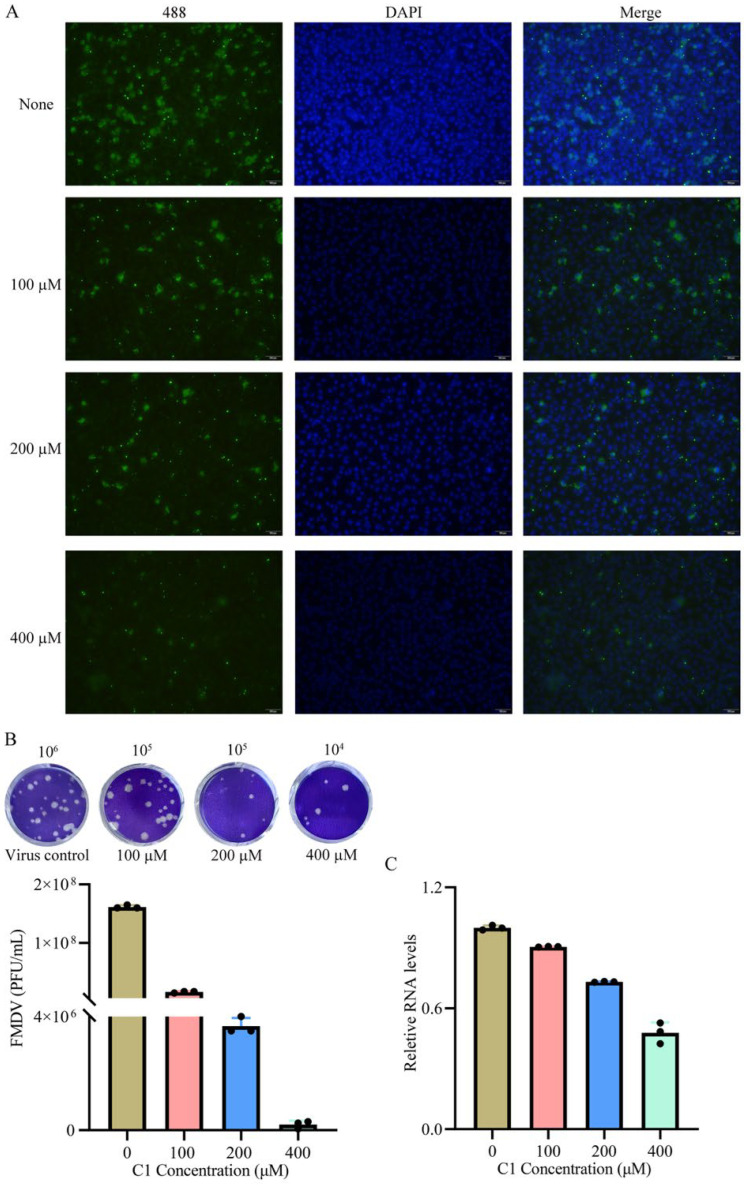
Effect of compound 2-MPO on FMDV replication in BHK-21 cells. (**A**) BHK-21 cells were inoculated with FMDV and treated with different concentrations of 2-MPO (100, 200, and 400 μM). For the indirect immunofluorescence assay, the primary antibody used was the rabbit anti-FMDV polyclonal antibody, detected with Alexa Fluor 488-conjugated goat anti-rabbit IgG secondary antibody (emission: 519 nm). All experiments were performed in triplicate. Nuclei were counterstained with DAPI. Scale bars: 100 μm. (**B**) The 2-MPO inhibited FMDV culture supernatant titers were determined by the plaque assay. (**C**) Viruses in infected cells treated with different concentrations of the compound (2-MPO) was detected by RT-qPCR.

**Figure 7:**
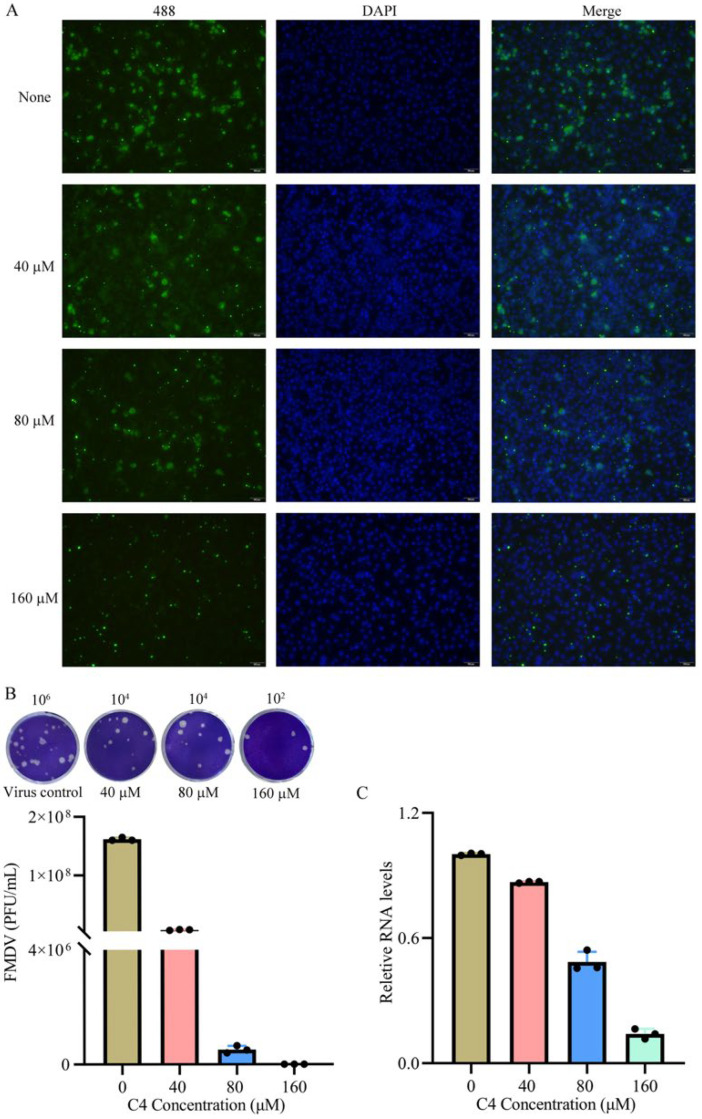
Effect of MPPI compound on FMDV replication in BHK-21 cells. (**A**) BHK-21 cells were inoculated with FMDV and treated with different concentrations of MPPI (40, 80, and 160 μM). For the indirect immunofluorescence assay, the primary antibody used was rabbit anti-FMDV polyclonal antibody, detected with Alexa Fluor 488-conjugated goat anti-rabbit IgG secondary antibody (emission: 519 nm). All experiments were performed in triplicate. Nuclei were counterstained with DAPI. Scale bars: 100 μm. (**B**) MPPI inhibited FMDV culture supernatant titers were determined by the plaque assay. (**C**) Viruses in infected cells treated with different concentrations of compounds (MPPI) were detected by RT-qPCR.

Guided by antiviral drug development criteria, five low-cytotoxicity compounds (high CC_50_ values) were selected for cellular-level antiviral efficacy screening. Indirect immunofluorescence assays revealed concentration-dependent antiviral activity (100 μM) across all candidates, with 2-MPO and MPPI demonstrating superior viral replication suppression (Figure S3). Dose–response analyses showed divergent potency: compared with the untreated control group, treatment with 2-MPO at 100 μM reduced viral plaque formation by 90.2%, with the inhibitory efficacy increasing to 97.3% at 200 μM and reaching 99.8% at 400 μM (*p* < 0.001), demonstrating a significant dose-dependent suppression of FMDV replication (Figure 6A,B). Furthermore, compound MPPI exhibited superior inhibitory efficacy compared with 2-MPO across the tested concentrations, achieving a 99.7% inhibition rate at 80 nΜ (Figure 7A,B). Reverse transcription quantitative PCR (RT-qPCR) analysis further validated that MPPI significantly outperformed 2-MPO in suppressing the FMDV RNA levels (Figure 6C and Figure 7C). These orthogonal evaluations establish MPPI as the most potent FMDV inhibitor identified through 2C helicase-targeted screening.

## 4. Discussion

FMDV causes FMD, a highly contagious infection affecting cloven-hoofed livestock with limited zoonotic risks [25,26]. FMDV’s seven serotypes lack cross-protective immunity, and antigenic variability among subtypes reduces vaccine efficacy against heterologous strains [27]. The World Organization for Animal Health (WOAH) categorizes FMD as a priority-listed disease due to its rapid transmission, persistent antigenic evolution, and socioeconomic impacts arising from trade restrictions, livestock mortality, and costly containment measures [26]. Given the limitations of existing vaccines in addressing antigenic diversity, developing broad-spectrum antiviral therapies against FMDV represents a critical unmet need in veterinary medicine. Current research on FMDV antiviral drugs has predominantly focused on the nonstructural proteins 3C^pro^ and 3D^pol^ [28,29,30,31], whereas investigations into 2C helicase as a therapeutic target have been sparse. This study identified small-molecule inhibitors targeting FMDV 2C helicase activity, a key viral replication mechanism, to suppress FMDV proliferation.

The FMDV 2C protein is essential for viral genome replication and progeny production within *Picornaviridae* [5,7,11]. As the most conserved nonstructural protein in *Picornaviridae*, 2C’s evolutionary conservation positions it as a prime target for broad-spectrum antiviral design [4,7,32]. We developed a FRET-based helicase assay and HTS platform to screen 4424 low-MW compounds for 2C inhibition. Low-molecular-weight (Low-MW) compounds enhance accessibility to cryptic binding pockets but limit multipoint interactions, often yielding suboptimal initial affinities [33,34]. These molecules improve the hit rates in HTS due to favorable properties and enable medicinal chemistry optimization for nanomolar affinity leads [20]. However, their limited heavy atom content inherently restricts the formation of multipoint intermolecular interactions, often resulting in suboptimal binding affinities during the initial screening phases. Elevated Mg^2+^ concentrations were identified as essential cofactors for FMDV 2C helicase activity in vitro (Figure 1B), mirroring the ATP-independent dsDNA unwinding functionality observed in EV71 and CVB3 2C homologs [9,10,11]. Notably, our optimized FRET-based assay enhanced dsDNA strand separation efficiency by eliminating competitive trapping strands, a methodological advancement enabling the precise monitoring of helicase kinetics (Figure 1F). Mechanistic analyses suggest that 2C’s intrinsic ribonuclease activity and ssDNA binding specificity preferentially redirect its enzymatic activity toward single-stranded substrates under competitive conditions, thereby attenuating dsDNA processing capacity [8,11].

The identified compounds selectively inhibit FMDV 2C helicase activity through direct protein–ligand interactions, as evidenced by the dose-dependent binding curves (Figure 3A and Figure S2). The binding assays demonstrated a significantly reduced affinity for the N207A mutant, confirming interactions with the conserved catalytic residues (Figure 3B and Figure S2). Molecular docking revealed that all compounds formed specific interaction bonds with the 2C active site, occupying similar binding pockets (Figure 3). Lineweaver–Burk kinetics analysis revealed competitive, noncompetitive, and mixed-type inhibition modes (Figure 4). Noncompetitive inhibition typically involves allosteric effects rather than direct active site binding. The low molecular weight of these compounds enhances access to cryptic pockets, enabling dual active site/allosteric modulation [34,35]. Due to the saturated substrate conditions in native-PAGE/FRET assays, precise IC_50_ determination was limited; however, the dose–response profiles confirmed micromolar viral suppression (Figures S3–S5). Guanidine hydrochloride (GuHCl), a known picornaviral 2C allosteric inhibitor, suppresses helicase and ATPase activities via conformational changes [9,36]. Our screening identified compounds 2-MPO and MPPI as mixed-type inhibitors that exhibit dual competitive and noncompetitive binding modalities toward FMDV 2C, coupled with low cytotoxicity profiles and superior therapeutic indices in viral suppression assays (Figure 4 and Figure 6). This mixed inhibition mechanism suggests the simultaneous engagement of catalytic and allosteric sites, potentially overcoming single-mode limitations. Such dual-target engagement likely underpins their broad-spectrum activity against diverse picornaviruses.

Novel pyrazolo [1,5-a] pyridine hybrid derivatives demonstrate promising broad-spectrum anti-enteroviral activity [37,38]. The MPPI small-molecule compound features an indole core sequentially fused with pyrazine and pyrimidine rings, bearing a methoxy substituent at the C11-position of the indole ring system. Similar to pyrazolo-pyridine hybrids, MPPI adopts a fused polycyclic heterocyclic framework. Its planar or semi-rigid conformation enables efficient insertion into viral protein hydrophobic pockets, enhancing binding affinity via π-π stacking and hydrogen bonding. Despite differences in ring composition, MPPI shares key design principles with pyrazolo-pyridines including a polyheterocyclic scaffold, viral protein-targeting strategies, and structure–activity relationship optimization. The intricate fused-ring system of MPPI represents a three-dimensional topological expansion of pyrazolo-pyridine scaffolds, aiming to amplify antiviral efficacy and broad-spectrum activity through increased heteroatom density and conformational rigidity.

**Figure 8:**
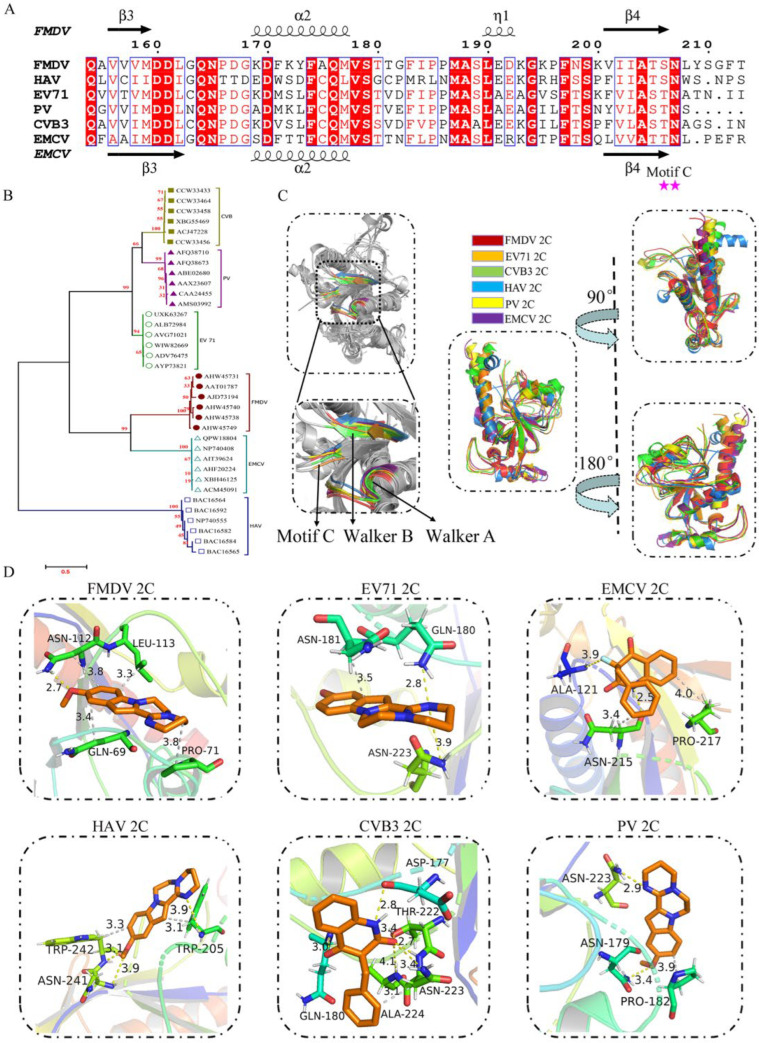
Based on picornavirus 2C amino acid sequence and structure analysis. (**A**) Amino acid sequence alignment analysis based on the structural analysis of the picornavirus 2C protein. 2C helicase key structural domain Motif C is marked with a red star. Blue boxes indicate that all amino acids within the same column exhibit a high degree of conservation. (**B**) Analysis of the amino acid genetic evolutionary tree of some picornavirus 2C proteins. (**C**) Structural comparison of FMDV 2C (red) with EV 71 (orange), CVB3 (green), HAV (blue), PV (yellow), and EMCV (purple). **Left**: The gray structure illustrates the spatial arrangement of Walker A, Walker B, and Motif C within the structural context of picornavirus 2C. **Upper right side**: The overlapping structure is rotated 90° clockwise on the Y-axis. **Lower right side**: The overlapping structure is rotated 180° clockwise on the Y-axis. (**D**) FMDV, EV 71, CVB3, HAV, PV, and EMCV 2C structures with the molecular docking results for C4 compounds. The brownish-yellow portion shows small molecule compounds with binding amino acid residue sites where the yellow bonds are hydrogen bonds and the gray bonds are hydrophobic interaction bonds.

Structure-guided sequence alignment identified universally conserved ATPase motifs across taxa, with Motif C showing the highest conservation (Figure 8A). Phylogenetic analysis revealed high sequence conservation within genera for 2C proteins, despite the C-terminal α-helical conformational divergence that parallels evolutionary divergence (Figure 8B). Furthermore, the 2C structures of six picornaviruses exhibited high structural superimposition, with the Walker A, Walker B, and Motif C domains overlapping and clustered in close proximity, thereby forming a large solvent-accessible pocket (Figure 8C). Notably, the picornavirus inhibitor MPPI demonstrated broad-spectrum potential through multi-residue interactions (≥3 residues) with 2C homologs from EV71, CVB3, HAV, PV, and EMCV (Figure 8D), exhibiting a favorable binding free energy (Δ*G* < −3.29 kcal/mol) stabilized by ≥2 hydrogen bonds and 1 hydrophobic interaction per complex. These findings suggest that FMDV 2C-targeted inhibitors like MPPI may exhibit cross-viral inhibition through conserved ATPase Motif C engagement, positioning them as promising broad-spectrum picornavirus candidates. Moreover, the specificity of MPPI for FMDV 2C necessitates a comprehensive evaluation to exclude off-target interactions with host AAA+ ATPases or unrelated viral proteases, which could compromise therapeutic safety.

Despite the comprehensive scope of our study, several limitations remain in the following aspects. (1) The exclusive reliance on the BHK-21 cell line, despite its utility in FMDV replication studies, introduces potential biases due to inter-lineage heterogeneity (e.g., variations in membrane receptor profiles, innate immune signaling cascades). Such variability may obscure compound-specific antiviral mechanisms or overestimate target specificity. (2) The absence of general protease inhibitors as experimental controls restricts mechanistic comparisons, potentially confounding the interpretation of compound specificity within a standardized pharmacological framework. (3) While in vitro antiviral activity against FMDV has been robustly demonstrated, critical gaps persist in validating in vivo efficacy (e.g., pharmacokinetics and toxicity in animal models) and broad-spectrum potential against other clinically relevant picornaviruses (e.g., enteroviruses, hepatoviruses). To address these limitations, priority should be given to: (i) validating findings in physiologically relevant systems such as primary porcine cells or FMDV-challenged animal models; (ii) optimizing structure-activity relationship (SAR) optimization to enhance potency and selectivity; and (iii) incorporating orthogonal controls to rigorously dissect target engagement.

## 5. Conclusions

In conclusion, we developed and validated a mechanism-driven HTS platform targeting FMDV 2C helicase activity. Our structure-guided approach identified MPPI as a broad-spectrum antiviral agent exhibiting micromolar-range inhibition of 2C helicase function. These findings provide a chemical framework for developing pan-picornaviral therapeutics through selective helicase inhibition, demonstrating the feasibility of targeting conserved 2C helicase domains for disease intervention in other picornaviruses.

## Figures and Tables

**Table 1:** Detailed information on six small molecule compounds.

Compound (Acronym)	Specs ID-Number	Name	Structure	InChIKeyTM	Molecular Formula
C1 (2-MPO)	AC-907/25014127	2-methylpyridine 1-oxide	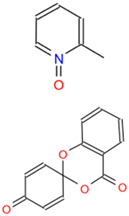	CFZKDDTWZYUZKS-UHFFFAOYSA-N	C_6_H_7_NO
C2 (5-TzS^−^)	AO-090/25092001	1H-1,2,3-triazole-5-thiolate	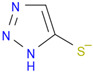	LLCOQBODWBFTDD-UHFFFAOYSA-M	C_2_H_2_N_3_S^−^
C3 (2-PyOH)	AC-907/25014059	pyridin-2-ol	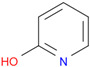	UBQKCCHYAOITMY-UHFFFAOYSA-N	C_5_H_5_NO
C4 (MPPI)	AO-082/13829008	11-methoxy-3,4,6,7-tetrahydro-2H-pyrimido[2′,1′:3,4]pyrazino[1,2-a]indole	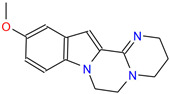	RPYFWWHSPMJDNM-UHFFFAOYSA-N	C_15_H_17_N_3_O
C5 (DCMQ)	AE-406/41056091	3,3-dichloro-8-methoxy-2,4(1H,3H)-quinolinedione	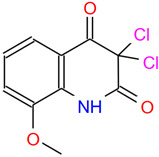	QUECMMKDBHBZHZ-UHFFFAOYSA-N	C_10_H_7_Cl_2_NO_3_
C6 (Spiro-BD-CHD-dione)	AN-970/40920757	Spiro (4H-[1,3]benzodioxine-2,4′-[2,5]cyclohexadiene)-1′,4-dione	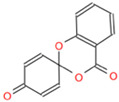	URIYZXDBDJZQMY-UHFFFAOYSA-N	C_13_H_8_O_4_

## Data Availability

The authors state that all data necessary for confirming the conclusions presented in this article are represented fully within the article or can be provided by the authors upon request.

## Supplementary Materials

The following supporting information can be downloaded at: https://www.mdpi.com/article/10.3390/v17060785/s1, Figure S1: 2D molecular docking; Figure S2: Changes in *T_m_* values for different concentrations of compounds interacting with 2C and the N207A mutant; Figure S3: Virus in infected cells that had been treated by compounds (C1–C5, 100 μM) was detected by Alexa Fluor 488-conjugated goat anti-rabbit IgG; Figure S4: 2-MPO and MPPI were tested in viral plaque reduction assays at varying concentrations; Figure S5: The inhibitory effects of C1–C6 small-molecule compounds on 2C helicase activity across varying concentrations.

## Abbreviations

The following abbreviations are used in this manuscript:

FMDV	Foot-and-mouth disease virus
FMD	Foot-and-mouth disease
FRET	Fluorescence resonance energy transfer
HTS	High-throughput screening
nanoDSF	Differential scanning fluorimetry
ORF	Open reading frame
NSP	Nonstructural protein
Arg-finger	Arginine finger
HAV	Hepatitis A virus
EV71	Enterovirus 71
CVB3/5	Coxsackievirus B3/5
PBL	Pocket-binding loop
PV	Poliovirus
EMCV	Encephalomyocarditis virus
IFA	Immunofluorescence
RT	Room temperature
DAPI	4′,6-Diamidino-2-phenylindole
GuHCl	Guanidine hydrochloride

## References

[1] Arzt J., Sanderson M.W., Stenfeldt C. (2024). Foot-and-Mouth Disease. Vet. Clin. North America. Food Anim. Pract..

[2] Grubman M.J., Baxt B. (2004). Foot-and-mouth disease. Clin. Microbiol. Rev..

[3] Gao Y., Sun S.Q., Guo H.C. (2016). Biological function of Foot-and-mouth disease virus non-structural proteins and non-coding elements. Virol. J..

[4] Norder H., De Palma A.M., Selisko B., Costenaro L., Papageorgiou N., Arnan C., Coutard B., Lantez V., De Lamballerie X., Baronti C. (2011). Picornavirus non-structural proteins as targets for new anti-virals with broad activity. Antivir. Res..

[5] Sweeney T.R., Cisnetto V., Bose D., Bailey M., Wilson J.R., Zhang X., Belsham G.J., Curry S. (2010). Foot-and-mouth disease virus 2C is a hexameric AAA+ protein with a coordinated ATP hydrolysis mechanism. J. Biol. Chem..

[6] Hurdiss D.L., El Kazzi P., Bauer L., Papageorgiou N., Ferron F.P., Donselaar T., van Vliet A.L.W., Shamorkina T.M., Snijder J., Canard B. (2022). Fluoxetine targets an allosteric site in the enterovirus 2C AAA+ ATPase and stabilizes a ring-shaped hexameric complex. Sci. Adv..

[7] Zhang C., Yang F., Wojdyla J.A., Qin B., Zhang W., Zheng M., Cao W., Wang M., Gao X., Zheng H. (2022). An anti-picornaviral strategy based on the crystal structure of foot-and-mouth disease virus 2C protein. Cell Rep..

[8] Chen P., Wojdyla J.A., Colasanti O., Li Z., Qin B., Wang M., Lohmann V., Cui S. (2022). Biochemical and structural characterization of hepatitis A virus 2C reveals an unusual ribonuclease activity on single-stranded RNA. Nucleic Acids Res..

[9] Xia H., Wang P., Wang G.C., Yang J., Sun X., Wu W., Qiu Y., Shu T., Zhao X., Yin L. (2015). Human Enterovirus Nonstructural Protein 2CATPase Functions as Both an RNA Helicase and ATP-Independent RNA Chaperone. PLoS Pathog..

[10] Chen Z., Xiong X., Li Y., Huang M., Ren Y., Wu D., Qiu Y., Chen M., Shu T., Zhou X. (2022). The nonstructural protein 2C of Coxsackie B virus has RNA helicase and chaperoning activities. Virol. Sin..

[11] Zhou S., Liu N., Tian Y., Pan H., Han Y., Li Z., Zhang J., Guan S., Chen H., Song Y. (2024). Enzymatic characterization and dominant sites of foot-and-mouth disease virus 2C protein. Heliyon.

[12] Guan H., Tian J., Qin B., Wojdyla J.A., Wang B., Zhao Z., Wang M., Cui S. (2017). Crystal structure of 2C helicase from enterovirus 71. Sci. Adv..

[13] Laufman O., Perrino J., Andino R. (2019). Viral Generated Inter-Organelle Contacts Redirect Lipid Flux for Genome Replication. Cell.

[14] Tang J., Abdullah S.W., Li P., Wu J., Pei C., Mu S., Wang Y., Sun S., Guo H. (2022). Heat Shock Protein 60 Is Involved in Viral Replication Complex Formation and Facilitates Foot and Mouth Virus Replication by Stabilizing Viral Nonstructural Proteins 3A and 2C. mBio.

[15] Guan H., Tian J., Zhang C., Qin B., Cui S. (2018). Crystal structure of a soluble fragment of poliovirus 2CATPase. PLoS Pathog..

[16] He Q.Y., Zhao H.F., Meng L., Geng Z., Gao Z.Q., Qi X.Y., Dong Y.H., Zhang H. (2024). A cardioviral 2C-ATP complex structure reveals the essential role of a conserved arginine in regulation of cardioviral 2C activity. J. Virol..

[17] Lv B., Yuan Y., Yang Z., Wang X., Hu J., Sun Y., Du H., Liu X., Duan H., Ding R. (2024). Stearoyl coenzyme A desaturase 1 (SCD1) regulates foot-and-mouth disease virus replication by modulating host cell lipid metabolism and viral protein 2C-mediated replication complex formation. J. Virol..

[18] Cui Z., Liu J., Xie C., Wang T., Sun P., Wang J., Li J., Li G., Qiu J., Zhang Y. (2024). High-throughput screening unveils nitazoxanide as a potent PRRSV inhibitor by targeting NMRAL1. Nat. Commun..

[19] Meyer C., Garzia A., Miller M.W., Huggins D.J., Myers R.W., Hoffmann H.H., Ashbrook A.W., Jannath S.Y., Liverton N., Kargman S. (2025). Small-molecule inhibition of SARS-CoV-2 NSP14 RNA cap methyltransferase. Nature.

[20] Miao J., Yuan H., Rao J., Zou J., Yang K., Peng G., Cao S., Chen H., Song Y. (2022). Identification of a small compound that specifically inhibits Zika virus in vitro and in vivo by targeting the NS2B-NS3 protease. Antivir. Res..

[21] Azzam T., Du J.J., Flowers M.W., Ali A.V., Hunn J.C., Vijayvargiya N., Knagaram R., Bogacz M., Maravillas K.E., Sastre D.E. (2024). Combinatorially restricted computational design of protein-protein interfaces to produce IgG heterodimers. Sci. Adv..

[22] Bailly M., Mieczkowski C., Juan V., Metwally E., Tomazela D., Baker J., Uchida M., Kofman E., Raoufi F., Motlagh S. (2020). Predicting Antibody Developability Profiles Through Early Stage Discovery Screening. mAbs.

[23] Yang R., Zhang Y., Geng B., Tian Y., Tian W., Zou Y., Chen H., Chen J. (2024). Fluorescence labeling-based differential scanning fluorimetry, an effective method for protein thermal stability and protein-compound binding analysis. Int. J. Biol. Macromol..

[24] Zeng J., Weissmann F., Bertolin A.P., Posse V., Canal B., Ulferts R., Wu M., Harvey R., Hussain S., Milligan J.C. (2021). Identifying SARS-CoV-2 antiviral compounds by screening for small molecule inhibitors of nsp13 helicase. Biochem. J..

[25] Jamal S.M., Belsham G.J. (2013). Foot-and-mouth disease: Past, present and future. Vet. Res..

[26] Ren X., Li P., Li X., Qian P. (2024). Epidemiological and genetic characteristics of foot-and-mouth disease virus in China from 2010 to 2022. Virology.

[27] Lu Z., Yu S., Wang W., Chen W., Wang X., Wu K., Li X., Fan S., Ding H., Yi L. (2022). Development of Foot-and-Mouth Disease Vaccines in Recent Years. Vaccines.

[28] Theerawatanasirikul S., Lueangaramkul V., Pantanam A., Mana N., Semkum P., Lekcharoensuk P. (2023). Small Molecules Targeting 3C Protease Inhibit FMDV Replication and Exhibit Virucidal Effect in Cell-Based Assays. Viruses.

[29] Kim Y., Pool E., Kim E., Dampalla C.S., Nguyen H.N., Johnson D.K., Lovell S., Groutas W.C., Chang K.O. (2024). Potent small molecule inhibitors against the 3C protease of foot-and-mouth disease virus. Microbiol. Spectr..

[30] Theerawatanasirikul S., Thangthamniyom N., Kuo C.J., Semkum P., Phecharat N., Chankeeree P., Lekcharoensuk P. (2021). Natural Phytochemicals, Luteolin and Isoginkgetin, Inhibit 3C Protease and Infection of FMDV, In Silico and In Vitro. Viruses.

[31] Mana N., Theerawatanasirikul S., Semkum P., Lekcharoensuk P. (2024). Naturally Derived Terpenoids Targeting the 3D(pol) of Foot-and-Mouth Disease Virus: An Integrated In Silico and In Vitro Investigation. Viruses.

[32] Zhang X., Paget M., Wang C., Zhu Z., Zheng H. (2020). Innate immune evasion by picornaviruses. Eur. J. Immunol..

[33] Chen H., Zhou X., Wang A., Zheng Y., Gao Y., Zhou J. (2015). Evolutions in fragment-based drug design: The deconstruction-reconstruction approach. Drug Discov. Today.

[34] Kirsch P., Hartman A.M., Hirsch A.K.H., Empting M. (2019). Concepts and Core Principles of Fragment-Based Drug Design. Molecules.

[35] Jhoti H., Williams G., Rees D.C., Murray C.W. (2013). The ‘rule of three’ for fragment-based drug discovery: Where are we now?. Nat. reviews. Drug Discov..

[36] Pfister T., Wimmer E. (1999). Characterization of the nucleoside triphosphatase activity of poliovirus protein 2C reveals a mechanism by which guanidine inhibits poliovirus replication. J. Biol. Chem..

[37] Hu Y., Kitamura N., Musharrafieh R., Wang J. (2021). Discovery of Potent and Broad-Spectrum Pyrazolopyridine-Containing Antivirals against Enteroviruses D68, A71, and Coxsackievirus B3 by Targeting the Viral 2C Protein. J. Med. Chem..

[38] Xing Y., Zuo J., Krogstad P., Jung M.E. (2018). Synthesis and Structure-Activity Relationship (SAR) Studies of Novel Pyrazolopyridine Derivatives as Inhibitors of Enterovirus Replication. J. Med. Chem..

