# Supplementary material for: Discovery of Small Molecules Against Foot-and-Mouth Disease Virus Replication by Targeting 2C Helicase Activity
Source: Viruses. 2025 May 29;17(6):785. doi: 10.3390/v17060785 (PMC12197642; doi:10.3390/v17060785)
Supplement: Supplementary file 1 [file viruses-17-00785-s001.zip › viruses-3614518-supplementary.pdf]

Discovery of Small Molecules Against Foot-And-Mouth Disease Virus Replication by Targeting 2C Helicase Activity

Saisai Zhou [1,2], Suyu Mu [3], Shuqi Yu [1,2], Yang Tian [1,2], Sijia Lu [1,2], Zhen Li [1,2], Hao Wu [1,2], Jiaying Zhao [1,2], Huanchun Chen [1,2], Shiqi Sun [3] and Yunfeng Song [1,2,*]

1 State Key Laboratory of Agricultural Microbiology, Huazhong Agricultural University, Wuhan 430070, China; zss2021302010119@webmail.hzau.edu.cn (S.Z.); yushuqi@webmail.hzau.edu.cn (S.Y.); tian_1995@webmail.hzau.edu.cn (Y.T.); lsj20010712@163.com (S.L.); swanlizen@webmail.hzau.edu.cn (Z.L.); 2023302010068@webmail.hzau.edu.cn (H.W.); z_jia_y@webmail.hzau.edu.cn (J.Z.); chenhch@mail.hzau.edu.cn (H.C.)

2 College of Veterinary Medicine, Huazhong Agricultural University, Wuhan 430070, China

3 Lanzhou Veterinary Research Institute, Chinese Academy of Agricultural Sciences, Lanzhou 730046, China; mutou6633937@126.com (S.M.); sunshiqi@caas.cn (S.S.)

* Correspondence: syf@mail.hzau.edu.cn

Supporting Information

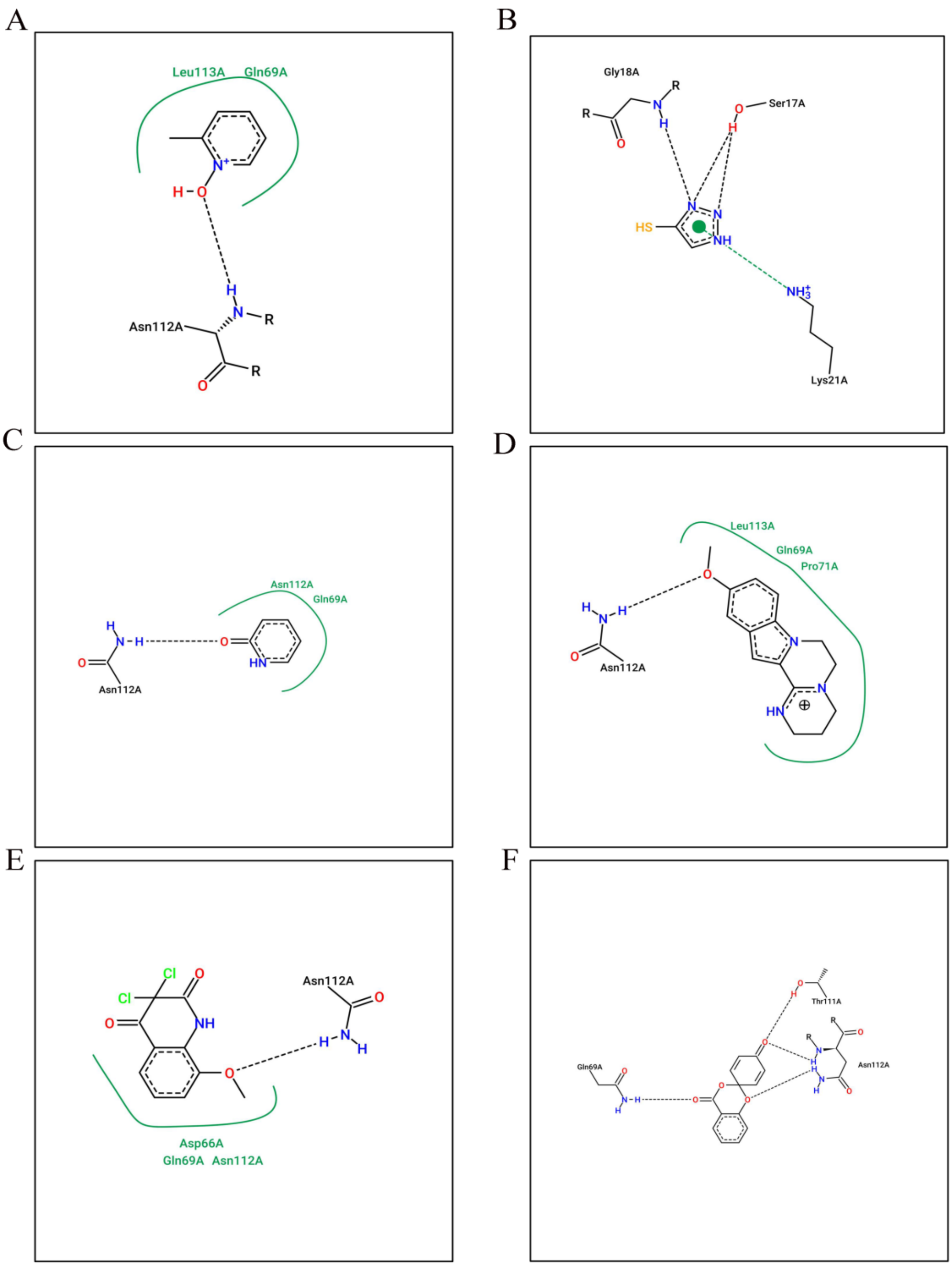

Figure S1. The 2D molecular docking. (**A**) 2D analysis of molecular docking between compound 2-MPO and the 2C protein structure. (**B**) 2D analysis of molecular docking between compound 5-TzS^{-} and the 2C protein structure. (**C**) 2D analysis of molecular docking between compound 2-PyOH and the 2C protein structure. (**D**) 2D analysis of molecular docking between compound MPPI and the 2C protein structure. (**E**) 2D analysis of molecular docking between compound DCMQ and the 2C protein structure. (**F**) 2D analysis of molecular docking between compound spiro-BD-CHD-dione and the 2C protein structure.

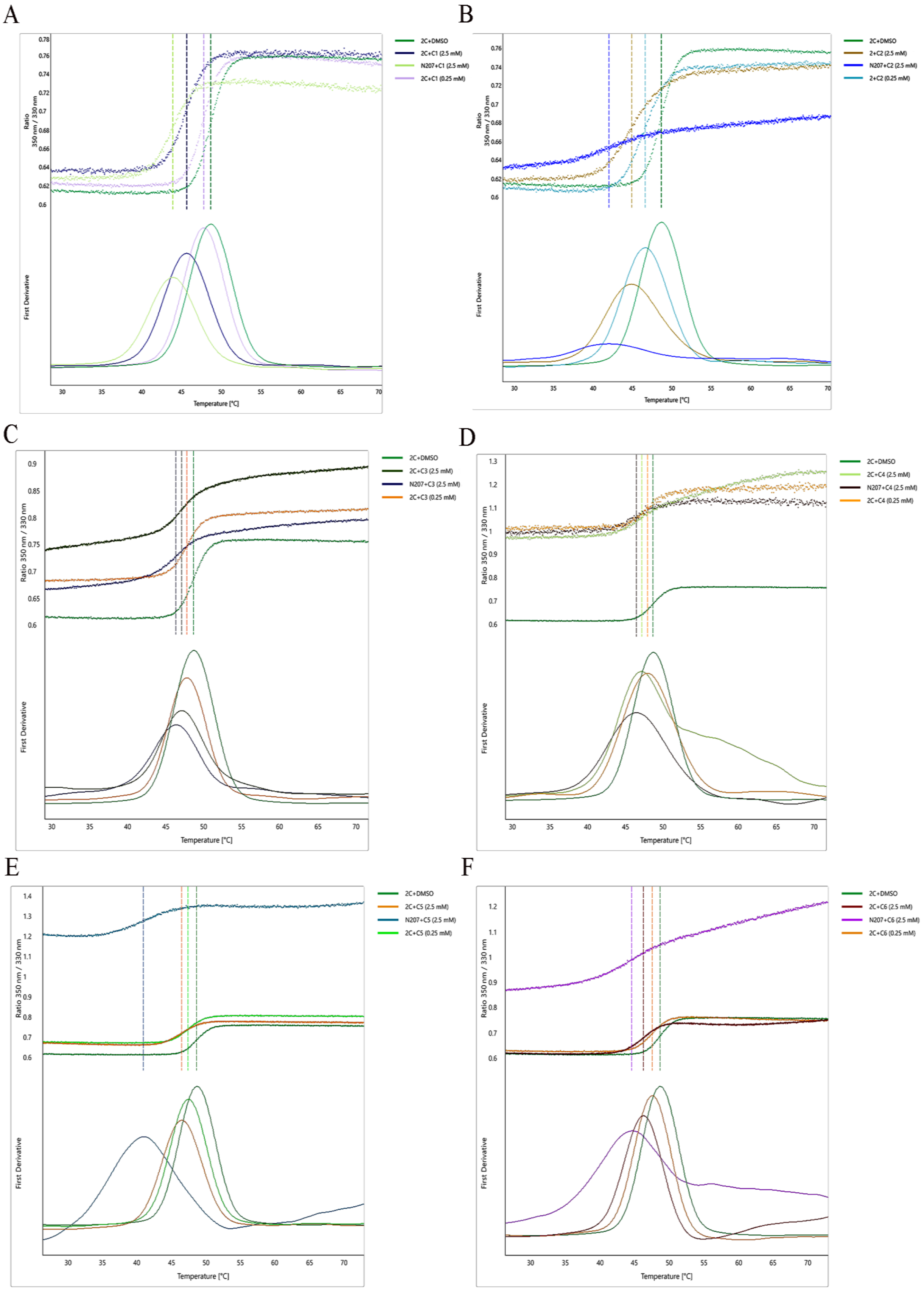
A
2C+DMSO
2C+C1 (2.5 mM)
N207+C1 (2.5 mM)
2C+C1 (0.25 mM)
Ratio 350 nm / 330 nm
First Derivative
Temperature [°C]
B
2C+DMSO
2+C2 (2.5 mM)
N207+C2 (2.5 mM)
2+C2 (0.25 mM)
Ratio 350 nm / 330 nm
First Derivative
Temperature [°C]
C
2C+DMSO
2C+C3 (2.5 mM)
N207+C3 (2.5 mM)
2C+C3 (0.25 mM)
Ratio 350 nm / 330 nm
First Derivative
Temperature [°C]
D
2C+DMSO
2C+C4 (2.5 mM)
N207+C4 (2.5 mM)
2C+C4 (0.25 mM)
Ratio 350 nm / 330 nm
First Derivative
Temperature [°C]
E
2C+DMSO
2C+C5 (2.5 mM)
N207+C5 (2.5 mM)
2C+C5 (0.25 mM)
Ratio 350 nm / 330 nm
First Derivative
Temperature [°C]
F
2C+DMSO
2C+C6 (2.5 mM)
N207+C6 (2.5 mM)
2C+C6 (0.25 mM)
Ratio 350 nm / 330 nm
First Derivative
Temperature [°C]

Figure S2. Changes in Tm values for different concentrations of compounds interacting with 2C and N207A mutant. (**A-F**) The upper panel of each graph represents the 350 nm/330 nm fluorescence ratio; the lower panel displays the first derivative of the ratio. (**A**) Comparison of Tm values between 2C and the N207A mutant protein in the presence of compound 2-MPO at 2.5 mM concentration. (**B**) Comparison of Tm values between 2C and the N207A mutant protein in the presence of compound 5-TzS^- at 2.5 mM concentration.
(**C**) Comparison of Tm values between 2C and the N207A mutant protein in the presence of compound 2-PyOH at 2.5 mM concentration. (**D**) Comparison of Tm values between 2C and the N207A mutant protein in the presence of compound MPPI at 2.5 mM concentration. (**E**) Comparison of Tm values between 2C and the N207A mutant protein in the presence of compound DCMQ at 2.5 mM concentration. (**F**) Comparison of Tm values between 2C and the N207A mutant protein in the presence of compound spiro-BD-CHD-dione at 2.5 mM concentration.

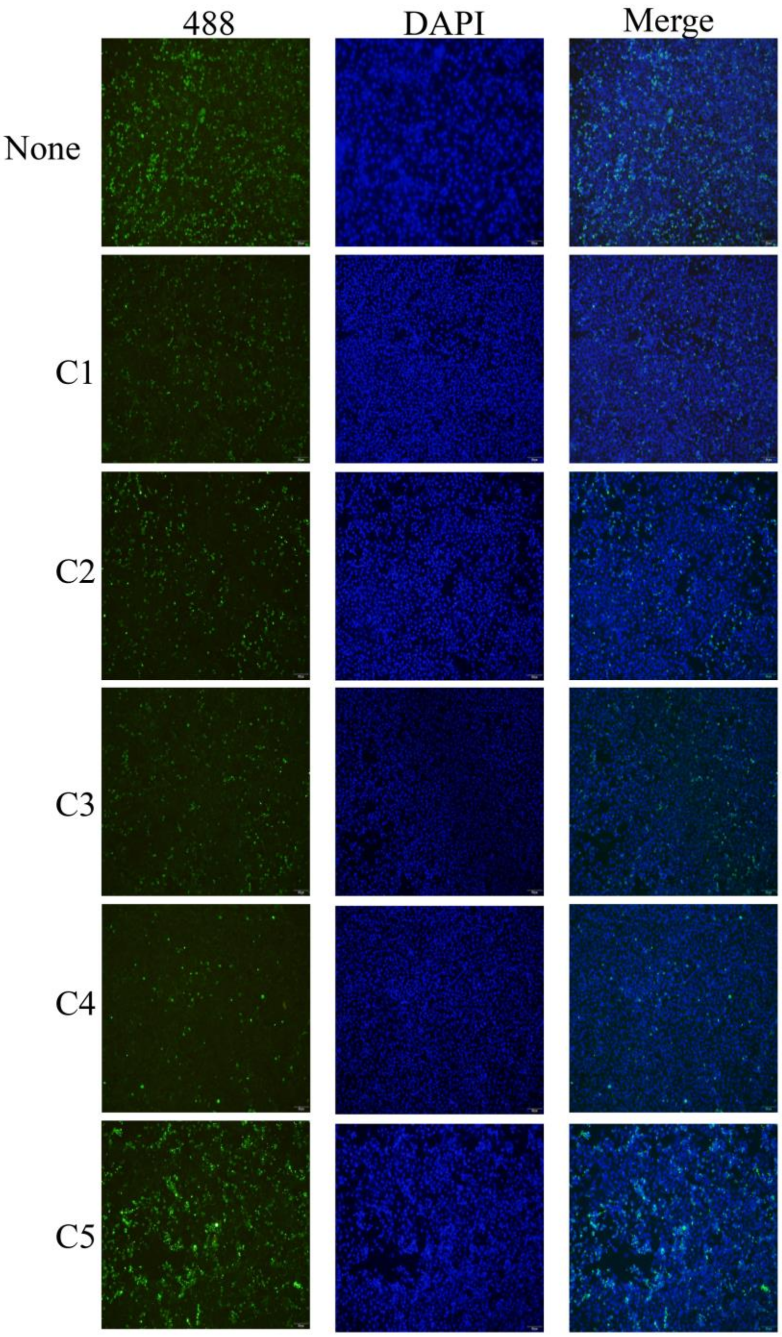

Figure S3. Virus in infected cells that had been treated by compounds (C1-C5, 100 μM) were detected by Alexa Fluor 488 -conjugated Goat Anti-Rabbit IgG. Cells were stained with DAPI (blue), and the FMDV protein was stained with Alexa Fluor 488-conjugated antibody (Green).

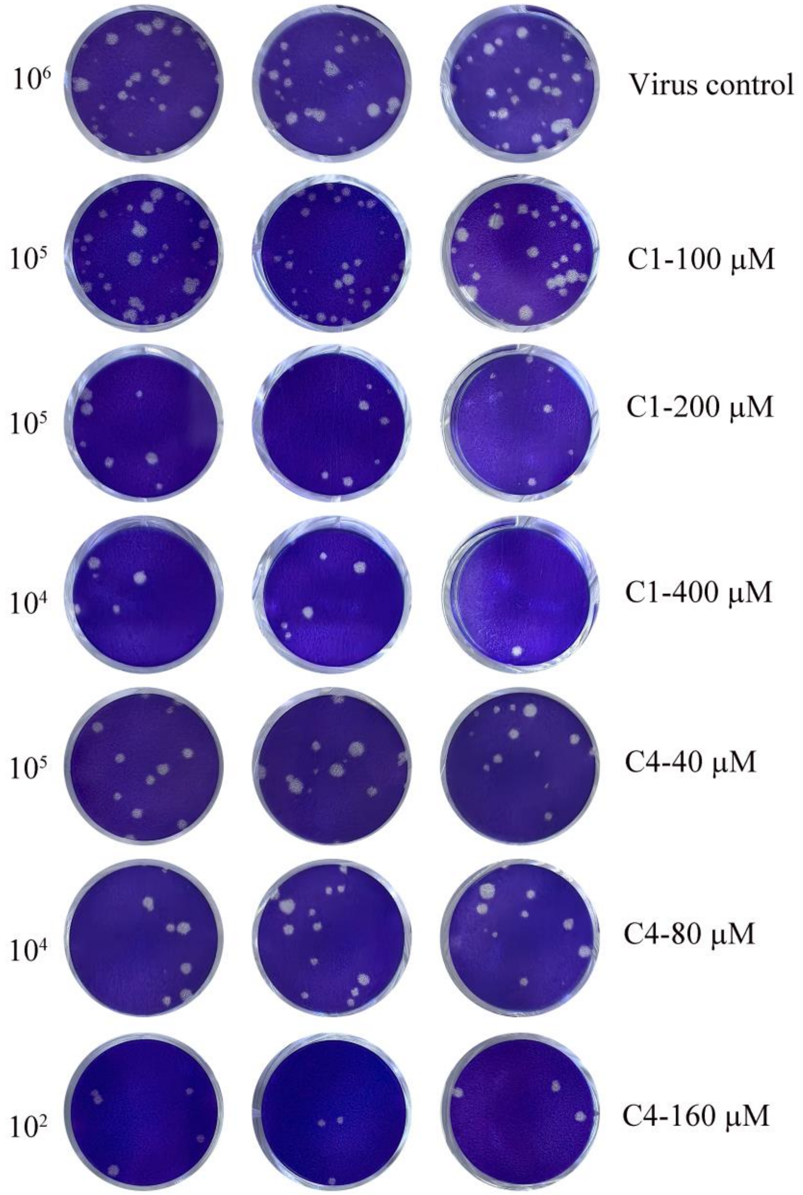

Figure S4. The 2-MPO and MPPI were tested in viral plaque reduction assays at varying concentrations. 2-MPO concentration gradients included 100, 200, and 400 μM, while MPPI gradients comprised 40, 80, and 160 μM. Each concentration was tested in three technical replicates.

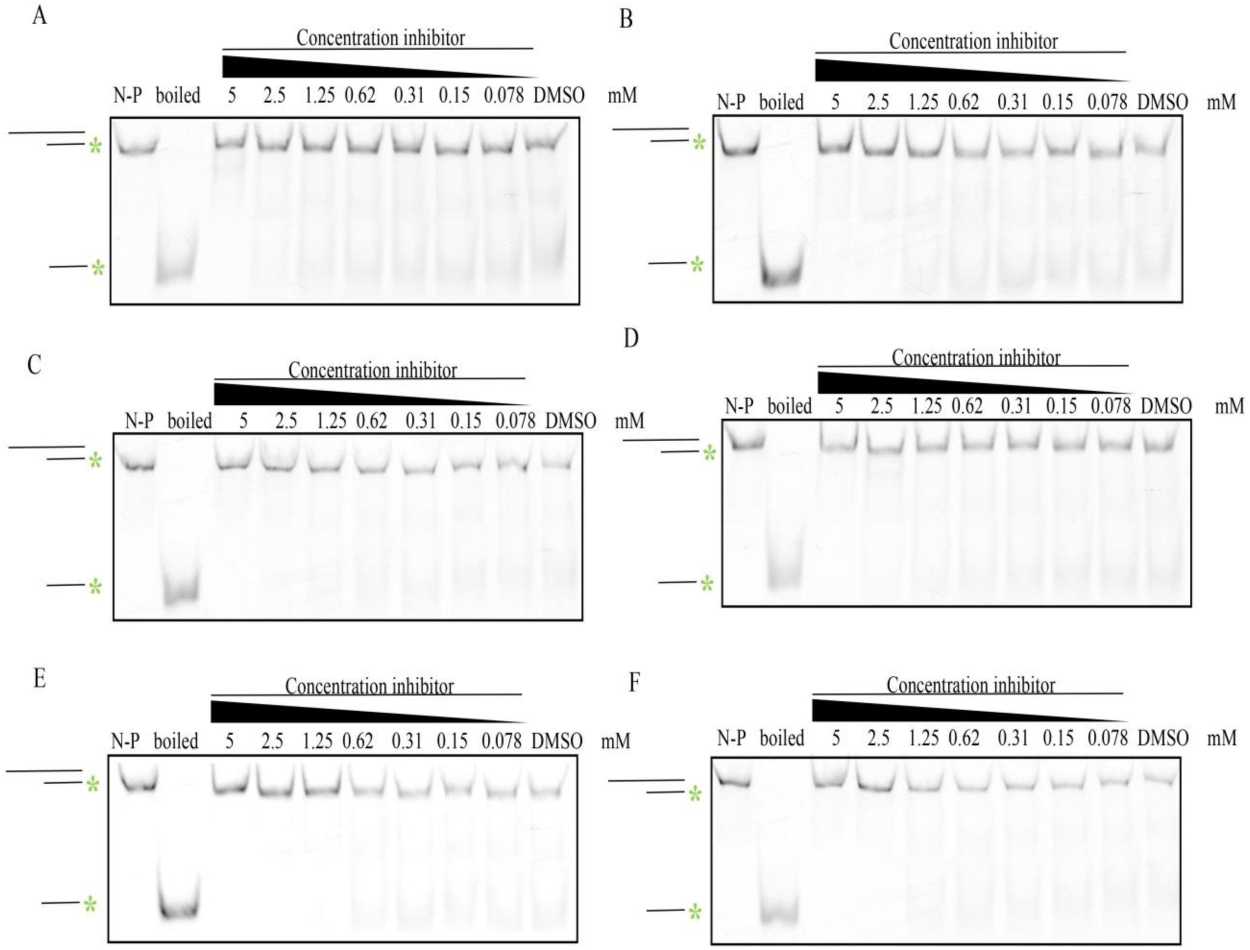

Figure S5. The inhibitory effects of C1-C6 small-molecule compounds on 2C helicase activity across varying concentrations. (**A-F**) Each compound was tested at seven concentration gradients. N-P: Represents the negative control where the dsRNA substrate remains non-unwound. boiled: Represents the positive control where dsRNA is denatured into single-stranded RNA (ssRNA) by boiling. DMSO: Represents the negative control. (**A**) The inhibitory effect of Compound 2-MPO on 2C helicase activity at various concentrations. (**B**) The inhibitory effect of Compound 5-TzS$^-$ on 2C helicase activity at various concentrations. (**C**) The inhibitory effect of Compound 2-PyOH on 2C helicase activity at various concentrations. (**D**) The inhibitory effect of Compound MPPI on 2C helicase activity at various concentrations. (**E**) The inhibitory effect of Compound DCMQ on 2C helicase activity at various concentrations. (**F**) The inhibitory effect of Compound spiro-BD-CHD-dione on 2C helicase activity at various concentrations.
